# The Attempt Was My Own! Suicide Attempt Survivors Respond to an Australian Community-Based Suicide Exposure Survey

**DOI:** 10.3390/ijerph16224549

**Published:** 2019-11-18

**Authors:** Myfanwy Maple, Kathy McKay, Rebecca Sanford

**Affiliations:** 1 School of Health, University of New England, Armidale 2350, Australia; 2 Department of Health Services Research, University of Liverpool, Liverpool L69 3GL, UK; KMcKay@Tavi-Port.nhs.uk; 3 Tavistock and Portman NHS Foundation Trust, London NW3 5BA, UK; 4 School of Social Work and Human Service, Thompson Rivers University, Kamloops, BC V2C 0C8, Canada; rsanford@tru.ca

**Keywords:** suicide attempt survivors, lived experience, suicide, suicide attempt, exposure

## Abstract

Those who attempt suicide have often been overlooked in the suicide prevention literature. Where stories of lived experience have been included, it is often from the perspectives of healthcare professionals who treat the physical and/or psychological impacts following an attempt, rather than firsthand accounts. Yet, the most intimate insights of suicide are lost by not including the voices of those with lived experience of suicide attempt. Through an online, community-based, non-representative survey exploring the impact of exposure to suicide, a sub-sample of 88 participants responded who reported their exposure to suicide as being their own attempt. The survey covered demographic information, questions assessing exposure to suicide attempts and death, current global psychological distress via the Kessler Psychological Distress (K10) Scale, and short qualitative responses provided by 46 participants. The qualitative data was thematically analysed resulting in three themes; the way in which individuals experienced being suicidal; who they were able, or not, to disclose these intentions to—before and after their suicide attempt; and, how these people experienced the formal and informal health care supports available to them to assist with their suicidal crisis. This paper presents important findings from a sample of participants who are highly distressed, and have previously attempted to take their own lives. This adds depth to our understanding of lived experience of suicide attempt, issues associated with seeking appropriate support after suicide attempt, and also demonstrates a willingness of participants to share their stories, even in a study that did not explicitly target those with lived experience of suicide attempt. The need for consistent and compassionate mental health care after a suicide attempt is identified as a vital component of living well after a suicide attempt.

## 1. Introduction

In Australia over 3100 people died by suicide in 2017 [1] with the number of people attempting suicide thought to be at least twenty times the number of suicide deaths recorded [2]. While rates of suicide death can often be under-reported, suicide attempts are even more likely to be under-reported for several reasons. Firstly, not all people who attempt suicide seek medical attention, or even tell anyone around them, and so are not recorded in any formal system. Secondly, even when presented to a healthcare professional, a suicide attempt needs to be recognised as such in order to be recorded appropriately; some suicide attempts can be more easily “explained” as accidental than intentional. Thirdly, individuals themselves report a variety of reasons they may not disclose their attempt, including stigma [3]. Yet, understanding the experiences of those who attempt suicide is crucial in suicide prevention efforts as a prior suicide attempt is the single biggest predictor of future death by suicide [2]. 

In the burgeoning suicide prevention literature, the first-hand accounts of those who have attempted suicide have often been neglected. Yet where people with lived experience of suicide have been included, they offer enormous insight into what is experienced prior to their attempt, the feelings attached to survival and life after an attempt, and the types of support that were, or were not, appropriate and effective. Lakeman and Fitzgerald systematically reviewed the existing literature to determine how people lived with their suicide ideation, especially in terms of “recovering a desire to live” [4] (p. 115). They found that people struggled with suicide ideation, and often described disconnection from others and intense suffering. A more recent review highlighted the balance people had to make between the intense suffering of continuing suicidal ideation and hope for the future after surviving a suicide attempt [5]. The experiences of intense suffering described in both reviews were akin to Shneidman’s *psychache* [6], where emotional torment can overwhelm an individual, but here refer to feelings post suicide attempt.

A person’s intense suffering may not necessarily be alleviated if they survive the very act they intended to alleviate it. Indeed, in sharing his own suicidal experience, Webb states: “[T]here is no sense of failure quite like failing at suicide” [7] (p. 9). Similarly, in an earlier study, Chesley and Loring-McNulty reported that only 10 of the 50 participants in their study reported feeling happiness or relief immediately after surviving their suicide attempt [8]. The predominant feelings reported were negative including feeling sad, depressed, disappointed, empty, angry, embarrassed or ashamed. While these negative emotions had changed for many participants by the time of the participation in the study, to reports of feelings of gladness, gratefulness, and hope, over 30% of participants continued to feel negatively towards their survival. Webb emphasises that one of the most important aspects of appropriate support after a suicide attempt is to feel listened to and validated, thus enabling the individual the opportunity to share their experience in their own words [7], without rejection, denial or avoidance of the subject. Ghio and colleagues’ study charted participants’ tumultuous times both before and after their suicide attempt, punctuated by extreme psychological distress and a perceived inability to cope, coupled with feelings of helplessness, hopelessness, and a disconnection from society [9]. Similar experiences were also reported by Australian men in the time before their suicide attempt, where they isolated themselves and family felt disconnected from them [10]. These first-hand reports of the emotional turmoil and difficulty managing emotions prior to, during and after a suicide attempt are in stark contrast to care and support systems being delivered to assist with suicide ideation and attempt, which fundamentally require individuals (or their carers) to reach out for help during these periods of extreme distress. 

It is clear that help-seeking and accessing appropriate and effective support remains difficult for many people following a suicide attempt. In a study examining the lifetime prevalence of suicidal ideation and attempts within a Queensland sample of 11,572 respondents, approximately 4% had attempted suicide in their lifetime, while just over 20% had thought at some point that life was not worth living [11]. Of these two groups, less than half sought some form of professional help (37% and 42% respectively). While many participants did not seek help because they felt they did not need it (either physically or psychologically), other reasons were more in line with the stigma attached to suicide which compounds the difficulty in accessing appropriate and timely supports. Both groups gave examples of not seeking help for their suicide planning or attempt due to not wanting to be a burden, concern around how healthcare professionals might treat them, and worries about what others would think of them if their suicidality was discovered [11]. 

Determining what help is required, and then approaching and accessing help, can be challenging. The Care After A Suicide Attempt (CAASA) Study [12,13] examined how individuals navigated care systems post suicide attempt. In this study, mothers talked about advocating on behalf of their daughters following suicide attempts and reported finding appropriate support was a matter of luck as they had not always been taken seriously by healthcare professionals, which had, in one case, led to tragic consequences [12]. Indeed, this mix of luck and the need for advocacy was echoed among the adult participants who had attempted suicide [13]. Participants spoke about the importance of consistent care in living well after their suicide attempt, which was not always available, and the small kindnesses that made a real difference in the immediate aftermath [13]. Feeling able to take back control is also reported as being important in restoring hope for living [14]. Here, as in other studies [15,16], negative experiences in professional settings where people had felt judged, made them less likely to want to seek help in the future. Yet, while stigma, involuntary hospitalisation and medication were the most common barriers to disclosure, Hom and colleagues [17] reported that disclosures of suicide ideation (past and current) to health providers was more common than to family members. This may be linked to the quality of the therapeutic relationship, but it may also be linked to age. In addition to formal support, informal support may also be more relevant to younger people. Gair and Camilleri [18] found that adolescents and young people were at times more likely to seek help from family and friends, rather than through any formal channels. Ghio and colleagues [9] found a similar importance placed on family and friends, especially when professional support was lacking, which has been reported elsewhere for individuals across the lifespan [19]. 

Those who have survived their own suicide attempt(s) have much experience to inform current prevention and intervention programs in order to make them more accessible, appropriate and person-centred and can provide important insights into how systems can respond to their needs at times of life-threatening distress. The present study contributes to the growing body of literature by sharing the experiences of people who self-identified as having attempted suicide within a general community survey on suicide exposure. 

## 2. Materials and Methods 

An online survey was distributed between April and August 2016 by Suicide Prevention Australia through member organisations of the Australian National Coalition for Suicide Prevention mailing lists to obtain a snapshot of Australian community members’ exposure to suicide. Due to this method of recruitment, it is not possible to know the response rate, nor the reach of the invitation to participate in the research. Ethics approval was obtained through the University of New England (HE16-030). These sampling procedures resulted in a total of 3220 responses; the full methods and results are available elsewhere [20]. Questions in the survey specifically asked about exposure to another’s suicide attempt and/or death. While no specific question in the survey requested information regarding a participant’s own suicide attempt, participants who offered a response of “self” or a similar variation (e.g. me, myself, it was me, my own attempted suicide) when prompted to indicate their relationship to the person whose attempt affected them the most or when asked to provide an open-text response to complete the sentence about relationship to the person who attempted suicide, being “This person was my…”, indicated one’s own suicide attempt were coded in the “self” category (*n* = 76). A further 12 participants indicated that their own suicide attempt was an additional exposure to suicide (among other exposure/s), and were also included in the “self” category for the purpose of this study. This sub-sample (*n* = 88) of the main study is the focus of this manuscript. 

### 2.1. Measures

The online survey consisted of approximately 30 questions. All respondents were asked demographic items querying sex, geographic location, and age; questions assessing exposure to suicide attempts and suicide deaths; current global psychological distress via the Kessler Psychological Distress Scale (K10) [21]; and one short qualitative response. 

#### Kessler Psychological Distress Scale (K10)

The K10 has been used as a measure of global psychological distress in general populations with good reliability and internal consistency. The K10 includes Likert-scale items in which participants are asked to identify how often they have experienced the identified problem in the last 30 days. Symptoms include: tiredness, nervousness, hopelessness, restlessness, worthlessness, and depression. Response options include: none of the time (1), a little of the time (2), some of the time (3), most of the time (4), and all of the time (5). Scores on the K10 range from 10 to 50 with higher scores indicating greater levels of distress. The K10 has a Cronbach’s alpha of 0.93 [21]. In this sample Cronbach’s alpha was 0.956, indicating an acceptable level of reliability. 

### 2.2. Qualitative Data

Participants were also given the opportunity to provide additional qualitative information regarding their exposure to suicide, with a prompt simply asking if they wanted to share anything further about their experience, 46 participants provided responses to this open-ended question. Of those, six provided statements of thanks for the survey, while the remaining 40 provided further insight into their experience. There was no limit to the amount of text that could be entered and entries ranged from short statements, to longer paragraphs with significant details. An example of a short statement is: “suicidal thoughts and images are a part of daily life for me, i try to manage this the best i can but it is so hard,” while an example of a longer response is: “I was dead on arrival to hospital and in a coma for a week and on dialysis. I still feel guilty with the stigma attached to mental health and this needs to change. I’m currently in a psych hospital and people think I’m rocking in a straight jacket instead of just having a break and a medication check. This thinking needs to end before we can get anywhere with mental health care” with a few responses that were over 200 words. This data was thematically coded by the first author by reading and re-reading the data to determine inductive codes. A second independent coder (KM) then coded the data, which were then checked by the third author (RS). Any conflicts were discussed until consensus was achieved. These themes are reported below without editing for spelling or grammatical errors, with identifying information removed. Due to the nature of data collection being via an online survey, responses varied considerably in length and overlap between themes. Some open-ended responses were presented by the participant as full sentences, and even paragraphs, while others were disjointed comments. Thus, presented themes indicate groupings of like information to determine independent themes for discussion without artificially splitting into sub-themes. 

## 3. Results

### 3.1. Quantitative Findings

Just over half of the sample were located in metropolitan areas, with an additional one-quarter from regional locations, and the remaining from rural and remote areas of Australia. This sample included 70 women and 18 men who ranged in age from 18 to 69 (M = 42.94; SD = 13.43). 

When asked “what was your relationship to the person whose attempted suicide affected you most,” 88 people nominated their own suicide attempt as an exposure to suicide. They had also been exposed to multiple other suicide attempts, with 30% only being exposed to their own attempt, 23% being exposed to one other attempt, and a further 34% being exposed to up to five suicide attempts, the remainder (13%) being exposed to more than six attempts. When asked about close relationships with those who had attempted suicide, 44% reported one close attempt exposure (again being “self”), while 47% reported more than one close attempt exposure. Interestingly, 9% reported no close attempt exposures and, as this sub-sample is reporting on their own attempts, this may be due to the nature of the survey design which is explored in the limitations section. Three-quarters reported knowing at least one person who had died by suicide, with multiple exposures to suicide death being common (Range 1–10, M = 2.98; SD = 2.179). Current psychological distress among those reporting their own suicide attempt indicates that over half reported high (13.6%) to very high (38.6%) levels of distress with an average K10 score of 25.34 (SD = 10.59). See Table 1.

### 3.2. Qualitative Findings

Three themes emerged from the analysis of the qualitative data provided by 40 participants. Demographically, these participants were very similar to the whole sample of self reporters (see Table 1). The three themes were; 1. Being suicidal, 2. Talking—or not—about suicide, and 3. Experiences of the health care system. These themes are presented below, with quotes from participants used to illustrate these themes. Quotes are identified by sex of participant, age and geographic location. Unsurprisingly, the majority of the qualitative data related specifically to the questions in the survey around distress and exposure to suicide attempt and death. Participants were also asked about the health care access and support the person/s they knew who had attempted or died by suicide had previously received. These specific questions likely drove the responses drawn out as themes below.

#### 3.2.1. Being Suicidal

The first theme, being suicidal, is derived from the ways in which participants described their suicide experience(s). This theme explores the ways in which these participants narrated their experience with suicide. For many this was in the distant past, while for some this was very recent. For all those who made mention of their own experience of being suicidal, the situation was clearly remembered, regardless of time since the event/s. Some participants wrote about how they felt when they had been suicidal, where they had been experiencing severe psychological distress: 

*I had a reactive nervous breakdown age 23. I was in psychiatric hospitals for most of the next 2 1/2 years due to major depressive illness and suicide attempts… I also self-harmed by injuring myself and cutting. I also developed OCD. I have not attempted suicide for over 15 years. I have worked full time for over 14 years.* Woman, 43, regional.

Many participants struggled with continuing mental health issues even if they no longer felt suicidal. These experiences had also lasted since adolescence for some:

*My attempt was because of internalised homophobia following the breakdown of a same sex attracted relationship at the age of 19.* Man, 67, regional.

*I never attempted suicide since l was 15 years of age but have had periods of time with major depression.* Woman, 46, metropolitan.

For some, their suicide attempt was perceived to be related to prior exposure to suicide attempt or death: 

*My own attempt was later in the same year—maybe as a direct consequence.*

Woman, 67, metropolitan.

This exposure to another’s suicide death or attempt, or their own experience of suicide, consequently seemed to provide insight into how people become suicidal, and participants referred to how they could understand the emotional place that a suicidal person is at due to their own experience of being in the suicidal state: 

*I have made several attempts at suicide myself. I feel I now have a greater understanding of the helplessness and fear that people feel when they prepare to take that “final” step.*

Woman, 51, remote.

*When my mother died, I had already attempted suicide 3 times myself, so I could understand why she did it. I was not affected therefore, by the fact that it was suicide that was the cause of her death. I only had to cope with the typical grieving I felt.*

Man, 58, remote.

Participants also wrote about how they were currently feeling while reflecting on their past suicide attempt: 

*I attempted to kill myself at the age of 7 years of age by taking antihistamines and cough medicine. I wanted to take the abuse that I was experiencing to stop rather than die. At the age of 13, I tried to kill myself by riding bike into a car at an intersection, again wanting to escape the abuse. In my early 20’s while living with fellow nurses I had suicidal thoughts connected with the household breaking up and feeling rejected which played into many of the issues connected to me childhood. Since then I have had a lot of counselling which was painful and I was very depressed but I have not been suicidal since then.*

Woman, 58, metropolitan.

There was a strong thread of having to work hard with mental health professionals afterwards in order to get to reduce suicidal thinking and actions:

*I was in a very dark place when I attempted (about 8 years ago) felt hopeless, worthless etc but friend took me to hospital and I got treatment afterwards (antidepressants and psychological counselling).*

Woman, 54, regional.

Further, this participant added, feeling better did not necessarily mean that all issues were resolved, but were being managed:

*Still on low dose meds but feel completely different now.*

Woman, 54, regional.

With the insight and empathy gained from their own attempt, participants wrote about how they were now involved in work supporting other people who were facing similar issues to the ones they had survived. This work could be enormously complex and challenging, as one participant wrote about how all his efforts to save his son had not prevented his death by suicide: 

*My own attempt occurred on the 11th August 1985 by gunshot as a result of alcoholism and after 11 1/2 months of rehab I was able to work and look after two of my sons as a single parent. AA was also a life changer and the AA programme is still big part of my life today. The last 13 years of my working life was in a mental health rehab unit and I was able to identify and help many of the clients just from my own experience. As a result of this work it was doubly devastating to watch my 38-year-old son drug himself to the point of paranoid schizophrenia and, regardless of my and his own best efforts via clinical intervention, he end his own life.*

Man, 69, regional.

#### 3.2.2. Talking—or Not—about Suicide

The second theme highlights the ways in which participants spoke about telling others of their suicide attempt (at the time of the attempt or since) or made conscious decisions not to tell others about their suicide history. Participants who had been suicidal during high-school expressed how they had not told anyone about their feelings at that time: 

*For my own experience, I was exhibiting signs of self harm and suicidal ideation, but having been a teenager at the time, I myself didn’t know, and in particular, my friends didn’t know, that the signs signified increased risk for suicide. I was also not in a position to seek professional help, as I didn’t know where to turn to, and felt alone in my situation.*

Woman, 41, rural.

And there was a struggle to find the language, the expressions, the words to describe what was being experienced: 

*As for me. Without knowing a name for it yet, I was in a deep depression for most of high school (mostly thanks to my parents unhappy marriage and abusive (mostly verbal, some physical) father. I constantly had suicidal thoughts, yet people knew me as the smiling girl who was friends with everyone, always happy. No one knew of my suicidal thoughts as I told no one.*

Woman, 39, Metropolitan.

While some did not access support, or know how to identify their needs and consider support, others did have active supportive connections at the time of their suicide attempt. Nevertheless, these supports were not always protective, and suicidal thoughts and plans where not always shared with these health professionals. For example, this participant did not tell their therapist, whom they saw, on the day they attempted suicide: 

*I saw my therapist on the day that I did it. It was a split second thought & I acted on it. I planned my will & sent emails whilst I was under the influence of the drugs & alcohol. I don’t even drink alcohol. My family just doesn’t get it & I’m sure it has had a significant affect [sic] on my 2 teenage daughters.*

Woman, 53, Metropolitan.

When able to express their suicidal thinking, for some this led to safe outcomes both immediately as well as future preventative measure: 

*Recently suicidal, suicidal ideation. At one critical point was able to speak to psychiatrist who gave me options and again saved my life. Wanting to die, or not wanting to live is so consuming when left to feed upon itself.*

Woman, 44, Metropolitan.

*I am getting help from my GP and a PTSD counsellor. This is my second bout. The suicide attempt was 8 years ago. This time hopefully I will avoid that by recognising the need for help and reaching out for it.*

Woman, 60, regional.

Safety in who and how to talk about the attempt was important, and this could take a long time to develop trust in being able to discuss suicidal thoughts. This participant powerfully expressed this as a “confession” when finally able to discuss their suicide attempt, but in doing so also then managed to obtain the support needed to stay safe:

*I had my own suicide journey and admitted myself to hospital twice. On my second admission I “practiced” my method, but didn’t tell anyone about it. I confessed to my psychiatrist 2 years later and got the support I needed.*

Woman, 32, Metropolitan.

Indeed, the idea that suicidality has to be “confessed” to a mental health professional implies the participant was hesitant to discuss these inner thoughts and actions, perhaps due to a sense of stigma and shame around the experience, but in this case resulted in a delay to the assistance that she required. One participant wrote about now being able to tell their suicide story to strangers as a way to offer support to people who are suicide bereaved, and that this provides new meaning and purpose for their life: 

*My own suicidal state has had a huge impact on myself and my family and friends I meet people who are bereaved and I am able to share the nature of the illness and bring a measure of comfort to their unanswerable questions people don’t realise that although I survived, my previous life ended. A normal family life that has never been the same again.*

Woman, 49, Metropolitan.

Even while being able to support others, there is also a sense that this type of sharing can be difficult as surviving an attempt does not necessarily mean a person’s life goes back to “normal” or to the pre-suicide state. This theme, which explores the ways in which people are challenged to find their voice to tell their suicide story provides a richer picture of how people may or may not be able to ask for help at the time prior to, during or after a suicide attempt. 

#### 3.2.3. Mental Health Care Systems

The third theme refers to the ways in which participants spoke of their experience of the mental health care system and the people providing care within the system. Involvement with this system often occurred over long periods of time and is experienced through multiple contacts to move beyond the suicidality the person had experienced:

*I survived my suicide attempt and after nearly 6 years of hospitalisation, drug and group therapy and psychiatric treatment, I no longer have suicidal thought and have my depression under control.*

Man, 69, Metropolitan.

While some were able to access supports that lead to positive outcomes, at the time of participating others had not been able to access the support they felt they needed either at the time of their attempt or afterwards. There was dissatisfaction with the focus on current physical quick-fixes through medication rather than more holistic practice of understanding the needs of the individual, as this participant explained of her recent suicide attempt: 

*6 weeks ago my suicide attempt was not treated with the care that should have been provided at the hospital I was treated at. I was not offered an opportunity to talk to anyone. Until many days later. I have not had after care that I require because I went from NSW to QLD. I am and have been left to seek help on my own. I am on medication that is not being monitored by any medical practitioner. I don’t think I’m getting better. The system is not good enough.*

Woman, 45, Rural.

Others also spoke about a lack of consistent care and being medicated without monitoring: 

*My experience at public hospitals has been terrible! I wasn’t given help, they just drugged me up until I was a zombie in the corner.*

Woman, 32, Metropolitan.

This lack of appropriate and compassionate care had worn down one participant who felt they continued to be at risk of suicidality. They likened their experience to that of someone they had known who had eventually died by suicide from such neglect: 

*It has taken its toll on my own life as I feel exactly the same way he did [referring to a person who had died by suicide]. With what is happening to me now, I will be joining him very soon. I just can’t cope anymore and nobody seems to care.*

Man, 58, Metropolitan.

Empathetic, appropriate and timely care are vital to ensure that a person in suicidal crisis is able to access both medical and therapeutic assistance when needed. However, this is complicated by the earlier theme of not being able to talk about the suicide attempt at the time. 

## 4. Discussion

The individuals who chose to identify their exposure to suicide as their own provide intimate insight into the suicidal experience. Remarkably, given the survey did not expressly ask about own exposure, these individuals found a way in which to highlight their experience regardless. This sub-sample of 88 individuals reported a range of experiences with suicide exposure, including their own previous attempt/s. While the K10 is limited in measuring the global psychological distress of a participant over the past 30 days, this sub-sample had considerably higher distress overall than the sample as a whole [20]. The averages over the range of distress for the whole survey sample were 36% recorded as low, 26% moderate, 20% high, and 18% very high, with an overall mean score of 20 [20]. Yet, this sub-group of the same sample, being those reporting their own suicide attempts, had 40% in the very high range, with only 25% in the low range. Furthermore, when compared to a representative sample of the Australian population, where 68% score in the “low” range and only 4% score in the “very high” category [22], it is clear to see that, while causal inference is not possible with the limitations of the study design, it is notable that this group of individuals are very distressed. 

The factors influencing the way in which people described either talking about their desire to end their life—or not talking about it—are important to consider to better appreciate how psychoeducational activities beyond general help-seeking messages can be delivered. At present the majority of suicide prevention messages focus on improving help seeking behaviour among those at risk, and yet this data demonstrates that people who have attempted suicide describe being unable to find the words to explain to others what is happening in their inner worlds, to the point where one participant refers to “confessing” their attempt to their treating psychiatrist many years later. This supports the emerging literature on suicide disclosure, and the difficulties others have reported in voicing their suicidal intentions [3,23]. While participants reported difficulties in disclosing their suicide ideation and attempt to others at the time, here in an anonymous survey they did disclose their attempt exposure as their own even in the absence of specific questions to do so. This may indicate a higher motivation to disclose in some settings than has been previously reported. 

The other priority in contemporary suicide prevention is adequately equipping gate-keepers to identify those at risk, yet many of these participants also described their dissatisfaction with services they had engaged with. Feelings of not being valued, or being pushed through a system, are unlikely to provide the space in which an individual can explore their feelings and find ways in which to tell others of their suicidal thoughts or actions. Simultaneously, participants also spoke about the protective nature of being connected with appropriate services. For many, connection with services was identified as core to their ongoing recovery [3]. This supports Chesley and Loring-McNulty [8] who identified common reasons for people no longer attempting suicide, including: being linked in with a professional, a sense of self-empowerment, current life success (personal or professional), and a new outlook on life. Common coping strategies that were utilized by the suicide attempt survivors heavily revolved around communication and connection. Our findings support this notion, that is, being able to talk about suicide opens a conversation to assistance, and longer-term positive outcomes. 

Importantly, these written narratives from participants in this study indicate how a suicide attempt, no matter how far in the past, could continue to impact on a person’s life. Webb [7] argues that the very act of a suicide attempt kills part of the self—a person is never the same again. This suggests that any support being offered to someone who has attempted suicide may need to focus on living well after the attempt, and finding new meaning and purpose, rather than “getting over it” or going back to a place from prior to the attempt [4]. In this way, the idea of “getting over” suicide may place a person in a dangerous position where there is a sense of uncertainty or shame in not being back to “normal”, as one participant states “a normal family life that has never been the same again.” Professional practice that incorporates compassion for those who have attempted suicide, or are presently at risk of suicide attempt, such as the model suggested by Cole-King and colleagues [24] provides guidance on holistic and importantly, compassionate, care to appropriately support people in times of suicidal crisis so that concerns about stigma, involuntary hospital admission and the like do not prevent people from accessing professional support. 

For many of these participants, it had been lifesaving to be able to talk about their suicidal feelings, even as they struggled with the language around how they were feeling. Talking about suicide is complex and requires a safe space and the knowledge that there will be no judgment around how the story is told and what the person has experienced and undertaken. This finding supports those reported from the CAASA Study where negative experiences of healthcare made participants feel less inclined to seek help in the future [13] and, in one instance, lead to a suicide death [12]. Indeed, consistent care, where people were able to see the same healthcare professional for a stable period of time, made participants feel safer in talking about their suicidality and less worried about being judged. Importantly, Kapur and colleagues have linked stable mental health service provision to a reduction in suicide attempts and mortality [25].

We caution that the findings presented here are limited by the study design in that the survey was designed specifically to explore the extent of exposure to suicide in the Australian community through a non-representative survey design. The survey was designed to be brief and delivered online via distribution through key suicide prevention mailing lists and websites. The survey was designed without explicit question regarding one’s own suicide attempt. Thus, those included in this sub-sample analysis were creative in choosing to report on their own attempt in the absence of a specific question in this regard. Whether these participants are representative of all people who completed the survey and had previously attempted to take their own life, or the Australian population of suicide attempt survivors is unknown. Furthermore, the measure used to examine distress, the K10, only provides a picture of global distress over the preceding four weeks. The scores from this measure are not reported as causally related to the suicide attempt, and are only used to present overall distress among this sub-group of participants who reported their own attempt.

## 5. Conclusions

Within the field of suicidology first-hand accounts of living with suicide ideation and past attempts are only recently emerging in the literature and public sphere. Without a variety of perspectives presented, suicide attempt can be viewed as a homogenous experience. Yet, this data suggests a continuum of suicide activity, from one attempt where the issues preceding the event were resolved and the individual continues with their (albeit changed) life, through to people who live with active suicide ideation and planning every day of their lives. This variability must be acknowledged in suicide prevention activities to better understand the role of suicide in an individual’s life and that this may be a changeable state or may resolve. Attending to the difficulties individuals have in voicing their suicidal thoughts and actions to others, and the factors that influence this, is an important next step in better understanding and responding to those experiencing suicidal crises. Further, actively including opportunities for individuals to share their experiences, particularly in anonymous surveys may provide greater insight into the ways in which suicide is experienced across the lifespan, and may assist in designing health services and suicide prevention programs that are responsive to individuals needs over time. 

## Figures and Tables

**Table 1:** Demographic and suicide exposure information for participants (*n* = 88).

Characteristic	*n* (%) (Total Sample)	*n* (%) (Respondents with Qualitative Response Only)
Geographic Region		
Metropolitan	47 (53.4%)	23 (51.1%)
Regional	22 (25.0%)	14 (31.1%)
Rural	13 (14.8%)	6 (13.3%)
Remote	5 (5.7%)	2 (4.4%)
Sex		
Woman	70 (79.5%)	36 (78.3%)
Man	18 (20.5%)	10 (21.7%)
Age		
18–24	7 (8.0%)	2 (4.3%)
25–34	16 (18.2%)	6 (13.0%)
35–44	25 (28.4%)	13 (28.3%)
45–54	19 (21.6%)	12 (26.1%)
55–64	16 (18.2%)	9 (19.6%)
65+	4 (4.5%)	4 (8.7%)
How many people do you know who have attempted suicide?		
One	27 (30.7%)	14 (30.4%)
Two	20 (22.7%)	11 (23.9%)
Three to five	30 (34.0%)	15 (32.6%)
Six or more	11 (12.6%)	6 (13.0%)
How many people with whom you have had a close relationship have attempted suicide?		
No close exposure	8 (9.1%)	3 (6.5%)
One close suicide attempt exposure	39 (44.3%)	23 (50.0%)
Multiple close suicide attempt exposures	41 (46.6%)	20 (43.5%)
How many people do you know who have died by suicide?		
Zero	17 (27.0%)	11 (23.9%)
One	28 (44.4%)	11 (23.9%)
Two	11 (17.5%)	6 (13.0%)
Three to five	6 (9.5%)	14 (30.4%)
Six or more	1 (1.6%)	4 (8.7%)
How many people with whom you have had a close relationship have died by suicide?		
No suicide death exposure	25 (28.4%)	10 (21.7%)
No close exposure	17 (19.3%)	10 (21.7%)
One close suicide attempt exposure	28 (31.8%)	16 (34.8%)
Multiple close suicide attempt exposures	18 (20.5%)	10 (21.7%)
K10 Severity Level		
Low (10–15)	22 (25.9%)	11 (24.4%)
Moderate (16–21)	17 (20.0%)	9 (20.0%)
High (22–29)	12 (14.1%)	7 (15.6%)
Very high (30–50)	34 (40.0%)	18 (40.0%)
